# Medication Literacy in Chinese Patients with Stroke and Associated Factors: A Cross-Sectional Study

**DOI:** 10.3390/ijerph20010620

**Published:** 2022-12-29

**Authors:** Xiao Chang, Kai Wang, Yuting Wang, Houmian Tu, Guiping Gong, Haifeng Zhang

**Affiliations:** 1 The First Affiliated Hospital of Anhui Medical University, Hefei 230032, China; 2 School of Health Management, Anhui Medical University, Hefei 230032, China

**Keywords:** stroke, medication literacy, medication safety, prevention of stroke, questionnaire

## Abstract

In China, stroke is characterized by high incidence, recurrence, disability, economic burden, and mortality. Regular and effective medication therapy can reduce stroke recurrence. High medication literacy is vital for the success of tertiary prevention measures aimed at preventing recurrence and minimizing disability. A cross-sectional survey using a medication literacy questionnaire was conducted between January and May 2022 on 307 inpatients of a Class III Grade A hospital in Hefei, Anhui Province, China. The demographic and clinical data of the patients were obtained from medical records. The health literacy of the patients was moderate, with 36.8% exhibiting adequate medication literacy. Univariate analysis identified significant differences in the medication literacy of the patients, depending on education level, annual income, family history of stroke, number of health problems, age, daily medication times, and brain surgery history. Multiple regression analysis revealed that education level, annual income, family history of stroke, and number of health problems significantly influenced medication literacy. In patients with stroke who are older and have a low education level, more health problems, no history of surgery, or no family history of stroke or medication guidance, medication knowledge and attitude can be improved to enhance medication safety and guarantee tertiary-level prevention of stroke.

## 1. Introduction

China has approximately one-fifth of the world’s total population and is, therefore, the largest developing country. The number of patients with stroke aged ≥40 years has reached 13.18 million in China, ranking first worldwide [1]. According to a global burden of disease study published in 2019 [2], the overall lifetime risk of stroke in China was 39.9%, which was also the highest worldwide. Stroke is a major chronic non-communicable disease with high rates of incidence, recurrence, disability, and mortality, carrying a high economic burden. Stroke seriously endangers the health of the Chinese population and is the primary cause of death and disability in Chinese adults. With the acceleration of population aging and urbanization, the prevalence of risk factors for stroke is obvious, and the disease burden of patients is increasing [3].

For patients with stroke, regular and effective medication therapy is a key factor in reducing the recurrence rate of stroke events [4,5], regardless of whether endovascular intervention is performed. Sui et al. [3] showed that unplanned readmission of patients with stroke was related to unsafe medication behaviors, such as unauthorized withdrawal from or poor adherence to medication regimens. Only patients with good medication adherence can effectively control the disease and delay or reduce the occurrence of related complications. However, most patients with stroke already carry a heavy medication load because they suffer from one or more chronic primary diseases [6], such as hypertension, diabetes, and coronary heart disease, which require lifelong medication [7]. Moreover, most patients with stroke are older and have poor memory, weak self-care ability, take many types of medications, lack communication abilities, and suffer from emotional disorders that can endanger their health [8,9]. The behavior of patients toward medication, especially their adherence to a medication regimen, has become the focus of clinical treatment and nursing. Some scholars have studied patient adherence behavior from the perspective of medication literacy [10,11,12]. The level of patient medication literacy has a significant impact on safe medication use, and patients with low medication literacy are more likely to require emergency department visits and unplanned readmissions, and to have poor prognosis due to irrational medication use, as reported previously [13]. Therefore, higher medication literacy is crucial for establishing tertiary-level prevention of stroke through measures that reduce its recurrence and minimize disability.

Currently, there is no universal concept of medication literacy [14]. The term was first used in a government document by the Medicines Safety and Healthcare Products Regulatory Agency Committee in the United Kingdom, which defines medication literacy as “a range of skills needed to access, understand, and use information on medicines” [15]. Pouliot et al. [16] proposed an expert consensus on the concept of medication literacy, which refers to the ability of individuals to access, understand, communicate, calculate, and process specific medication information to make informed medication treatment and health decisions to achieve safe and effective medication use. Sauceda et al. [17] referred to the definition of health literacy from the National Library of Medicine of the United States, in which the concept of medication literacy is defined as “the ability of individuals to obtain and understand information about medications and use this information to use medications in a safe and appropriate manner.” In other words, medication literacy covers the knowledge, beliefs, skills, behavior, and other aspects of a patient’s ability to use medications. It not only involves medication regimen adherence and management but also covers the ability of patients to use medications safely.

Our study referred to published medication literacy scales and used a tertiary hospital in Anhui, China, as a research site to investigate the medication literacy of 307 patients with stroke. We aimed to understand the current situation and determine factors influencing the medication literacy of these Chinese patients with stroke and to provide a scientific basis for further guidance on safe medication for patients with stroke in clinical practice.

## 2. Materials and Methods

A cross-sectional survey was conducted from January to May 2022. A total of 307 inpatients with stroke in the department of neurology of a Class III Grade A hospital in Hefei, Anhui Province, China, were selected by the purposive sampling method to conduct a medication literacy questionnaire survey.

### 2.1. Subjects

Patients with stroke who met the following inclusion criteria were enrolled in our study: (1) meeting the diagnostic criteria of acute ischemic stroke [18], with diagnosis confirmed by computed tomography or magnetic resonance imaging of the head; (2) age, ≥18 years; (3) having basic communication skills and the ability to understand simple instructions; and (4) providing informed consent.

The exclusion criteria were (1) presence of unconsciousness or mental disorders; or (2) presence of severe heart failure, respiratory failure, tumors, and other end-stage conditions.

### 2.2. Sample Size Calculation

Sample size was calculated using the following equation:

N = Zα/2^2^π(1 − π)(DEFF)/d^2^, (1)

where α is the allowable error bound to 0.05, and the statistic Zα/2 equals 1.96. π is an estimate of the expected probability of occurrence, and d is the allowable absolute error level, which was deemed ±10%. DEFF (design effect) is the survey accuracy. In random sampling studies, DEFF was set to 1; in nonrandom sampling studies, DEFF values ranged from 1 to 3. According to the sampling method used in this study, the DEFF value was 3 [13]. The sample size was 279. The final sample size was 293, considering a 5% failure rate.

### 2.3. Data Collection

Data collection was completed by two clinical nurses with a master’s degree, who were professionally trained before data collection to ensure data rigor and scientific professionalism. The investigators selected subjects who met the inclusion criteria after obtaining approval from the ethics committee. Subjects were informed about the purpose and significance of the study and signed an informed consent form. Participants were asked to fill out a general demographic information questionnaire (including questions on age, sex, educational level, income, marital status, occupation, residence, medical insurance type, and medical history) and a medication literacy questionnaire. The questionnaires were completed and retrieved on site, and the investigators provided timely explanations for options that were difficult for the participants to understand and ensured that all other aspects of the data collection were consistent. After the data collection, the participants’ answers were checked for completeness. Participants were asked for reasons why some items were unanswered, and they were then requested to complete the questionnaire as much as possible.

### 2.4. Survey Tools

The general information questionnaire was a self-designed questionnaire, which included items on sex, age, educational level, marital status, residence, medical insurance type, income level, number of health problems, daily medication frequency, medication duration, medication type, and history of brain surgery. The Chinese version of the medication literacy tool was derived from Sauceda et al.’s [17] original assessment questionnaire in Spanish and English (MedLitRxSE), which was developed to assess the level of medication literacy in patients. The tool consisted of 14 items. Four scenarios of medication use were simulated during the application of the scale, including following the doctor’s advice for insulin injections, taking over-the-counter fever medicine for children, identifying the name of a prescription medicine and calculating the required dose, and understanding medication administration instructions. Scores were given on a 2-point scale out of a total of 14 points, with higher scores indicating better medication literacy in each subject. The overall Cronbach’s α coefficient of the scale was 0.81. The scale was translated into Chinese and applied [19].

### 2.5. Statistical Analysis

All data in this study were recorded by two authors and checked for errors. SPSS 26.0 (version 26.0, Chicago, IL, USA) software was used for statistical analyses. We examined the collected data and then collated and coded them. Categorical variables are reported as frequency and percentage, and medication literacy scores of patients are expressed as mean (standard deviation). MedLitRxSE scores show a non-normal distribution. Therefore, we used the Kruskal–Wallis H test for univariate analysis. The MedLitRxSE score was considered our primary parameter of interest. To explore the influence of candidate variables on the MedLitRxSE score, we carried out a multiple linear regression analysis. The MedLitRxSE score was defined as the dependent variable, whereas history of brain surgery and six other candidate factors (age, years of schooling, annual income, family history of stroke, health problems, and number of daily medications) were considered independent variables and were included in the model. The assignment of factors in the multiple linear regression model was as follows.

Age group: 18–44 years = 1, 45–59 years = 2, ≥60 years = 3; years of schooling: ≤6 = 1, 7–9 = 2, 10–12 = 3, 13–16 = 4, >16 = 5; annual income: ≤10,000/year = 1, 10,000–29,000/year = 2, 30,000–49,000/year = 3, 50,000–99,000/year = 4, ≥100,000/year = 5; family history of stroke: yes = 1, no = 2; number of health problems: 1 or below = 1, 2 = 2, 3 or above = 3; times of medication use per day: 1 or below = 1, 2 = 2, 3 or above = 3; and history of cerebrovascular surgery: yes = 1, no = 2.

We made the following analysis for the multivariate linear model, including the regression coefficient, independence test, multicollinearity test, and residual normality test. The significance criterion was α = 0.05; if *p* was <0.05, the result was considered statistically significant.

## 3. Results

### 3.1. General Information of the Subjects

A total of 321 patients with stroke were invited to participate in this study: nine patients refused to participate in the study and five patients did not complete the questionnaire, leaving 307 (95.6%) patients who partook in the final analysis. Among these 307 patients, there were 157 men (51.1%); mean age was 56.84 ± 10.22 years; 42 patients (13.7%) had a bachelor’s degree or above, accounting for 25.4%; 78 patients (25.4%) had an annual family income of more than 100,000 yuan; 265 patients (86.3%) were married; and 154 patients were retired. Furthermore, 121 patients (39.4%) had a new rural cooperative medical insurance, 138 patients (45.0%) had experienced a stroke <3 years before the study, 95 patients (30.9%) had taken medications for <3 years at the time of the study, 164 patients had a family history of stroke, and 176 patients (57.3%) had a history of brain surgery (Table 1).

### 3.2. Medication Literacy of Patients with Stroke

Table 2 shows the percentage of correct answers for each question in the four scenarios for the 307 patients with stroke. In brief, the mean score of medication literacy was 8.95 ± 3.49, and the top three percentages of correct answers were for questions 12 (80.1%), 13 (77.9%), and 11 (76.2%). The three lowest accuracy percentages were for questions 3 (48.5%), 2 (53.8%), and 5 (54.4%). Among the different scenarios, questions in scenario four had the highest percentage of correct answers and questions in scenario one had the lowest percentage of correct answers.

### 3.3. Univariate Analysis of Medication Literacy of Patients with Stroke

Table 3 presents the specific medication literacy scores in different groups of patients with stroke. Patients of different ages with different years of schooling, annual income, family history of stroke, health problems, daily medication times, and history of brain surgery had different medication literacy scores.

### 3.4. Multiple Linear Regression Analysis of Factors of Medication Literacy in Patients with Stroke

Table 4 shows the results of multiple regression analysis. Years of schooling, annual income, family history of stroke, and number of health problems were identified as factors influencing medication literacy.

## 4. Discussion

In this study, the medication literacy score of patients with stroke was 8.95 (3.49), which is moderate. Zheng’s survey of 425 outpatients in Changsha gave a similar score of 8.31 (3.47) [19]. According to the principle of the calculation formula of distinction degree, which is used in educational statistics, the scores were classified into three groups: inadequate literacy (<4), marginal literacy (4–10), and adequate literacy (>10) [19]. The proportion of patients with adequate, marginal, and inadequate medication literacy was 10.4%, 52.8%, and 36.8%, respectively. Table 2 shows the proportions of correct answers for each question of the medication literacy questionnaire survey. The top three correctly answered questions were questions 12, 13, and 11, indicating that patients with stroke could clearly identify the medication validity period and active ingredients, and could convert medication measurements. The three questions with the lowest accuracy rate were questions 3, 2, and 5, indicating that patients with stroke have relatively lesser understanding of the application of insulin pens and their medical consultation options. This result differs from those of other populations in previous studies, such as of patients with hypertension [20] or coronary heart disease [21], reflecting the uniqueness of patients with stroke. Most patients with stroke have hypertension, hyperlipidemia, diabetes, coronary heart disease, or other comorbidities. To combat the problems with high error rates, we should give timely medication guidance to patients with high blood sugar, including information on oral insulin time, drug dose, adverse reactions, and other precautions. For patients who need to use insulin pens, the way to use the pen should be demonstrated. The patient’s memorization can be deepened by means of video display, on-site demonstration, and written step display, so as to facilitate the use of insulin at home after discharge. To ensure good understanding of return visits at the time of discharge, inform the patient of the specific process for a return visit (including the department for registration, doctor recommendation, specific floor, and other details), in case of emergency.

In the univariate analysis, we found that medication literacy was significantly correlated with age, history of brain surgery, daily medication frequency, education level, annual income, family history of stroke, number of health problems, and other factors. In the multiple linear regression, we found that years of schooling, annual income, and family history of stroke had a positive relationship with medication literacy scores. Number of health problems was negatively correlated with medication literacy scores.

The medication literacy score of patients in the younger age group was higher, and the younger patients may have had higher questionnaire scores than the older ones because they had fewer health problems, higher literacy, and a more active mind. However, multi-factor regression analysis revealed that even factors such as education level and financial conditions, which were individually positively correlated with age, did not yield significant differences. Zheng’s survey found similar results [19].

The medication literacy score of patients with a history of brain surgery was significantly higher than that of patients without a history of brain surgery. Typically, patients who have undergone surgery have deeper awareness of the risk of a negative disease prognosis, and their family members and the patients themselves pay more attention to postoperative physical maintenance and medication. Furthermore, these patients are more familiar with the composition and usage of medications and exhibit higher adherence than those who have not undergone surgery. Therefore, patients with stroke who have a history of brain surgery generally show higher medication literacy.

It was found that the higher the frequency of daily medication, the lower the patient’s medication literacy score. More medication indicates a more serious patient condition, more patient frailty, and decreased self-management ability; therefore, patients in this category show lower medication literacy scores.

Medication literacy scores increased with increased educational level. Ma et al. [1] reported similar results in patients with hypertension. Patients with higher education levels generally pay more attention to disease progression and the medication process, and a higher education level can help patients to better understand medication-related information. Thus, in this study, these patients could answer the questions correctly even when they had no prior experience with the questionnaire. On the other hand, patients with lower education levels were found to have lower medication literacy scores, and they could only follow the treatment and guidance provided by medical staff.

Stroke is a chronic disease with a long-term course that requires long-term prevention of recurrence and rehabilitation. High-income patients with stroke are better able to pay attention to their quality of life after the illness than low-income patients, so these patients put greater emphasis on the method, measurement, composition, and other information accompanying medication use. Moreover, a multicenter study found that income level was positively correlated with disease outcome [22]. High-income groups can afford advanced medical treatment easily; for example, they actively use advanced treatment methods and purchase appropriate medications, indicating that they pay more attention to the details of the disease treatment and medication methods to improve the prognosis of the disease.

The presence of long-term illnesses, taking care of older family members with cerebral apoplexy, and having a better general knowledge of diseases and medication regimens will provide an increased understanding of stroke occurrence and development and assist in achieving more positive disease prognosis in families than otherwise. These behaviors are of fundamental importance for controlling the risk of stroke; at the same time, they encourage positive measures, such as the rational use of medications in accordance with a doctor’s advice, as well as seeking medical consultation for the timely control of the disease.

Common concomitant diseases in patients with stroke include hypertension, diabetes, coronary heart disease, and hyperlipidemia. These health problems may affect each other and become risk factors for stroke. This study showed that patients with stroke who had a higher number of health problems had lower medication literacy scores. First, we observed that the poorer the physical and mental health of patients suffering from multiple diseases, the lower the self-management ability and medication adherence, and the lower the psychological acceptance of medication. Second, patients with multiple health conditions may need to take multiple medications at the same time, and the frequency of daily medication administration will increase, which may lead to confusion about the role, usage, and dosage of medications.

Higher medication literacy contributes to medication safety and a more positive disease prognosis in patients with stroke, but our study results suggest that there is much room for improvement in the medication literacy of these patients. Our results also confirm the importance of medication literacy in patients with cerebral apoplexy. Literacy intervention groups provide a theoretical basis for the clinical need to focus on factors such as age, low level of education, low income, more health problems, absence of previous surgery, no family history of stroke, medication knowledge, attitude, and behavior to strengthen guidance and improve patient safety as far as the caregivers are concerned.

In terms of medication knowledge, the communication of medication knowledge among patients with stroke should be strengthened. The stroke medication knowledge manual comprises the instructions of all commonly used medications for stroke. While seeking medical treatment, patients are allowed to have full information regarding the principles, types, and names of medications, medication methods, precautions before and after medication, efficacy, and adverse reactions to medications administered by doctors. The manual focuses on the observation of changes in disease symptoms and the treatment of adverse reactions during medication, as well as on consolidating medication information after discharge.

In clinical care, nurses and doctors can use some communication skills to help patients with stroke develop a sense of faith and confidence in taking medication. Motivational interviewing [23,24] can be used to change the medication attitudes of patients with stroke. Empathy and other communication methods can be used by caregivers to communicate their understanding of the emotional and psychological needs of patients with stroke to relieve negative emotions toward medication and establish timely medication confidence.

In terms of medication behavior, patients with stroke and their families were guided to implement some easy exercises to improve medication safety. For example, they used high-quality cards to schedule the patient’s medication. The type, time, dose, and frequency of medication were recorded with eye-catching words to remind the patients of regular medication administration. Setting an alarm on a mobile phone for the patients to take their medication on time and regularly is another example of these exercises. Patients with regular daily activities were recommended to combine them with taking their daily medication. Patients were provided with medication-dispensing boxes and requested to place their daily morning, afternoon, and evening doses into boxes of different colors or to label each box with a different time to ensure the accuracy, frequency, and correct dosing of medication. The main caregiver for a patient with stroke should be empowered and informed to prompt and supervise the medication behavior of the patient, to ensure safe and punctual medication administration.

Through scientific and effective management and training methods, health administration departments can improve the level of medical services and health education of medical workers and effectively improve the level of medication literacy of patients. At the same time, governmental control and policies to help primary caregivers in providing sustainable self-medication guidance for patients with stroke and improve the tertiary stroke prevention system are needed.

Our study has some limitations. The participants of this study were from a single tertiary hospital. Although the patients came from different cities of Anhui Province, certain regional limitations that reflect their medication literacy differences still exist. Although some patients came from other provinces, statistical analysis of data from this subset of patients could not be carried out due to limitations on data volume. Second, this study was a cross-sectional survey and could not show changes in the medication literacy of patients after discharge. In addition, the overall sample size was limited; therefore, further studies are required to determine whether the findings from this population can be generalized to populations with stroke in other parts of China. Targeted intervention studies are expected to improve the medication literacy of patients with stroke and to follow them up after discharge.

Our study also has several strengths. First, to the best of our knowledge, this is the first survey on medication literacy among Chinese patients with stroke that has aimed to analyze its influencing factors. Second, the patients in this study presented with similar comorbidities, and the use of data from a tertiary hospital of which it can be believed that the diagnosis of stroke is accurate further strengthens the scientific nature of the study results. Finally, we identified some novel factors influencing medication literacy, such as family history of stroke, which was not reported in previous studies.

## 5. Conclusions

We found that the medication literacy of patients with stroke needs further improvement. Based on the main relevant factors noted in this study, including years of schooling, annual income, family history of stroke, and number of health problems, in targeting the inadequate literacy group, it is necessary to pay more attention to the exchange of medication information with inpatients with stroke, strengthen discharged patients’ follow-up, and improve patient medication safety.

## Figures and Tables

**Table 1:** Demographics of patients with stroke (N = 307).

Variable Name	Items	No. of Participants (N = 307)	Percentage (%)
Sex	Men	157	51.1
	Women	150	48.9
Age	18–44	38	12.4
	45–59	181	59.0
	≥60	88	28.7
Years of schooling	≤6	101	32.9
	7–9	103	33.6
	10–12	27	8.8
	13–16	34	11.1
	>16	42	13.7
Annual income (CNY)	<10,000/year	42	13.7
	10,000–29,000/year	98	31.9
	30,000–49,000/year	55	17.9
	50,000–99,000/year	34	11.1
	≥100,000/year	78	25.4
Marital status	Married	265	86.3
	Unmarried	42	13.7
Occupation	Occupied	72	23.5
	Jobless	81	26.4
	Retired	154	50.2
Habitation	Urban	188	61.2
	Rural	119	38.8
Type of medical insurance	New rural cooperative medical insurance	121	39.4
	Medical insurance for urban workers	57	18.6
	Medical insurance for urban residents	106	34.5
	Other	23	7.5
History of stroke	<3 years	138	45.0
	3–4 years	130	42.4
	5–9 years	34	11.1
	≥ 10 years	5	1.6
Years of medication use	<3 years	95	30.9
	3–4 years	121	39.4
	5–9 years	51	16.6
	≥10 years	40	13.0
Number of people living together	1	19	6.2
	2	143	46.6
	≥3	145	47.2
Number of health problems	1	112	36.5
	2	139	45.3
	≥3	56	18.2
Family history of stroke	Yes	164	53.4
	No	143	46.6
Times of medication use per day	1	49	16.0
	2	137	44.6
	≥3	121	39.4
Number of medications currently taken	1	45	14.7
	2	101	32.9
	≥3	161	52.4
History of cerebrovascular surgery	Yes	176	57.3
	No	131	42.7

CNY 1 = USD 0.1436.

**Table 2:** Medication literacy by case scenario for patients with stroke.

Case Scenario	Item	Number of Correct Participants	Percentage of Correct Answers
Case Scenario 1	Q1 According to the label, how many times per day should your mother inject the medicine?	172	56.0%
	Q2 Please show me how much medicine you should put into the syringe in the morning and mark it on the syringe.	165	53.8%
	Q3 According to the instruction, please tell me or point out the three parts of the body into which your mother could inject the medicine.	149	48.5%
	Q4 According to the instruction, please tell me what is the correct angle at which you should inject the medicine.	173	56.4%
	Q5 Looking at the prescription, if your mother’s medicine has run out, from whom should you get a new prescription?	167	54.4%
Case Scenario 2	Q6 Looking at the instructions on this box, how much of the medicine should you give to your niece?	212	69.1%
	Q7 If you know the dosage of medicine that your niece needs to take, please mark on the cup to which line you poured the medicine.	187	60.9%
	Q8 According to the directions, what is the maximum dosage your niece should take?	187	60.9%
Case Scenario 3	Q9 Looking at this prescription, what is the name of the medicine that you need to buy at the pharmacy?	192	62.5%
	Q10 According to the prescription, how many pills should you take?	197	64.2%
	Q11 How many boxes should you buy to obtain the correct amount of antibiotic required by the original prescription?	234	76.2%
Case Scenario 4	Q12 Looking at the box, when does the medicine go out of date?	246	80.1%
	Q13 According to the directions, what is or what are the active ingredient(s) of each pill?	239	77.9%
	Q14 Please look carefully at the box. For what reason should you stop taking the medicine?	228	74.3%

**Table 3:** Comparison of medication literacy scores in different groups of patients with stroke (N = 307).

Factor	Items	Mean (SD) of the Total Score	H	*p*
Sex	Men	8.92(3.31)	−0.126	0.900
	Women	8.98(3.67)		
Age	18–44	10.45(2.61)	8.913	<0.001
	45–59	9.19(3.47)		
	≥60	7.83(3.54)		
Years of schooling	≤6	7.83(3.15))	49.520	<0.001
	7–9	7.27(3.07)		
	10–12	9.26(1.83)		
	13–16	11.91(2.26)		
	>16	13.19(0.86)		
Annual income (CNY)	<10,000/year	6.29(2.76)	68.660	<0.001
	10,000–29,000/year	7.30(2.68)		
	30,000–49,000/year	7.65(3.42)		
	50,000–99,000/year	11.15(2.46)		
	≥100,000/year	12.44(1.17)		
Marital status	Married	8.91(3.54)	−0.519	0.604
	Unmarried	9.21(3.14)		
Occupation	Occupied	8.89(3.58)	0.067	0.936
	Retired	9.07(3.70)		
	Jobless	8.92(3.34)		
Habitation	Urban	8.82(3.33)	−0.691	0.490
	Rural	9.10(3.72)		
Type of medical insurance	New rural cooperative medical insurance	8.74(3.92)	1.790	0.149
	Medical insurance for urban workers	9.82(3.30)		
	Medical insurance for urban residents	8.92(3.22)		
	Other	8.13(2.22)		
History of stroke	<3 years	8.84(3.48)	1.053	0.369
	3–4 years	8.83(3.66)		
	5–9 years	9.59(2.91)		
	≥10 years	11.00(1.87)		
Years of medication use	<3 years	9.56(3.45)	2.506	0.059
	3–4 years	8.77(3.49)		
	5–9 years	8.00(3.52)		
	≥10 years	9.30(3.29)		
Number of people living together	1	8.37(3.27)	1.113	0.330
	2	8.73(3.38)		
	≥3	9.26(3.61)		
Number of health problems	1	11.96(1.72)	114.627	<0.001
	2	7.19(3.06)		
	≥3	7.32(3.01)		
Family history of stroke	No	7.49(3.31)	−8.833	<0.001
	Yes	10.64(2.87)		
Times of medication use per day	1	9.22(3.55)	3.391	0.035
	2	9.42(3.11)		
	≥3	8.32(3.79)		
Number of medications currently taken	1	9.11(3.37)	1.608	0.202
	2	9.41(3.40)		
	≥3	8.63(3.56)		
History of cerebrovascular surgery	No	7.82(3.27)	−7.141	<0.001
	Yes	10.48(3.18)		

**Table 4:** Results of multiple linear regression analysis of determinants of medication literacy in patients with stroke (N = 307).

Determinants	β	SE	T	*p*	95% CI	
Constant	4.745	0.974	4.873	<0.001	2.829	6.661
Age group	−0.024	0.213	−0.113	0.91	−0.444	0.396
Years of schooling	0.646	0.115	5.609	<0.001	0.42	0.873
Annual income	0.968	0.124	7.794	<0.001	0.723	1.212
Family history of stroke	1.233	0.276	4.465	<0.001	0.69	1.776
Times of medication use per day	−0.172	0.18	−0.958	0.339	−0.525	0.181
History of cerebrovascular surgery	0.427	0.28	1.524	0.129	−0.124	0.978
Number of health problems	−0.945	0.212	−4.462	<0.001	−1.362	−0.528

β: partial regression coefficient; SE: standard error. R^2^ = 0.624, F = 54.743, *p* < 0.001.

## Data Availability

Not applicable.
